# Supplementary material for: Complexome profiling of the Chlamydomonas psb28 mutant reveals TEF5 as an early PSII assembly factor
Source: Plant Cell. 2025 Mar 17;37(6):koaf055. doi: 10.1093/plcell/koaf055 (PMC12164590; doi:10.1093/plcell/koaf055)
Supplement: koaf055_Supplementary_Data [file koaf055_supplementary_data.zip › psb28_interactive.zip!/plots/intensity/qualityPlots/peptides/_Cre14.g633550.t1.2.html]

### Cre14.g633550.t1.2

Trivial name:   
SeqCoverage [median]: 33.80  
Mass: 13.66 kDa  
  
 

| Sequence | MissedCleavage | Mass [Da] | wtIntensity | muIntensity | Score |
| --- | --- | --- | --- | --- | --- |
| ASGPLLQQLAK | 0 | 1124.66 | 768.00 | 3878.26 | 122.28 |
| LAPVLEAAQPR | 0 | 1163.67 | 1932.89 | 2525.42 | 93.65 |
| SLETEGAQLASK | 0 | 1232.62 | 705.23 | 2497.62 | 113.69 |
| VNPQTAFLTR | 0 | 1145.62 | 1472.96 | 2065.66 | 85.68 |
|  |  |  | 4879.1 | 10967.0 |  |
